# Mr. Ring, in Reply to Dr. Adams

**Published:** 1805-02-01

**Authors:** John Ring

**Affiliations:** New Street, Hanover Square


### *To the Editors of the Medical and Physical Journal.*

GENTLEMEN,

SOME people are of opinion, that the subject of Vaccination is exhausted. To me, however, it appears, that the discussion of that subject has increased, is increasing, and ought not to be diminished, till the practice is universal, or the small-pox exterminated from the face of the earth.

On a former occasion I expressed a doubt, whether the secondary eruptions which occurred after the insertion of supposed vaccine virus, in the practice of Dr. Adams, when at Madeira, were of the vaccine kind. That doubt is not lessened by any thing which I have since read.

In the infancy of the practice it was generally believed, that the cow-pock was an eruptive disease. This was owing to the variolated atmosphere which many of the patients breathed; or to the introduction of the small-pox into the system by some other means, instead of the cow-pock. It is well known that this particularly occurred in the practice of Dr. Woodville; yet Dr. Adams tells us, that in this respect he is content to stand on the same ground with Dr. Woodville.

It was my painful lot to point out the true nature of those eruptions, and to prove that they were the real small-pox; not eruptions produced by the cow-pock, in consequence of the patient breathing a variolated atmosphere, as some gentlemen imagined. In proportion to the care taken to avoid all communication with the small-pox, those eruptions ceased; and medical practitioners in general now consider the cow-pock as a local disease. What those eruptions were which appeared in the practice of Dr. Adams, I must leave to others to determine.

Rashes of different kinds are frequent attendants on the cow-pock; but I have never yet seen a single instance of a secondary vaccine vesicle, that might not easily be accounted for, in consequence of a second inoculation with the nail of the patient. In very young infants, who are not able to scratch their arms, I have never seen a vesicle of this kind.

Dr. Adams's eruptions are the less likely to have been vaccine, from their having been observed, in some instances, where no sign of infection appeared at the place of insertion. Two such deviations from the usual law of nature require long experience to confirm them.

It

It is true I expressed a doubt concerning Dr. Adams's cases, when we met previous to his journey into Scotland. The same doubt was, and is still entertained, by those inoculators with whom I am acquainted. Not expecting to meet Dr. Adams, I had not for a considerable time read his description of those cases; I, therefore, in the course of conversation mentioned, that many eruptive cases had occurred in my own practice, and that of others, in the infancy of vaccination; and that those eruptions were now well known to have been variolous.

Dr. Adams has, undesignedly, made me commit a great blunder, by misquoting my words. He makes me say, that matter taken from such eruptions has produced the chicken-pox. It will, however, appear, by referring to my letter in the Medical and Physical Journal for October, that the eruptions to which I then alluded were those of the chicken-pox, not the small-pox.

Dr. Adams notices the candour and moderation of Mr. Cardan of Worcester; if he will inquire into the conduct of the practitioners in this metropolis upon similar occasions, he will find that he need not have travelled to Worcester for such examples.

Dr. Adams says, we ought always to give our antagonist credit for all the good intentions he professes; and that a very slight knowledge of the world will teach us, this is the readiest way to convince him. A very slight knowledge of the world, however, will teach us, how dangerous it is to trust to professions; and that it is not easy to convince a man of any truth, which stands in opposition to his interest. None are so blind as those who will not see.

Dr. Adams asserts, that if the favourers of vaccination should be ready to admit some of those motives suspected by Dr. Macdonald, its enemies will think still worse of a cause, which requires such insinuations to support it. If, however, Dr. Macdonald saw reason to suspect sinister motives in any antagonist, it was his duty to declare it. If his suspicions were ill-founded, his insinuations would rather injure himself than the practice he meant to defend.

I cannot but be rather surprized at Dr. Adams's opinion, that a defect of evidence or irregularity, even when acknowledged, reflects on Mr. Goldson the highest honour; since there were other witnesses who could have corrected any misrepresentation.

Dr. Adams pronounces an eulogium on Mr. Goldson's veracity, and affirms, that when he was anxious to discredit vaccination, he did not strain his evidence. To me, however, however, and to many others, he appears to have strained his evidence; some instances of which are to be found in my answer to his pamphlet, and others in that of Dr. Macdonald. Had he strained it more, he would have been liable to detection; and have stamped an indelible stigma on the cause he meant to support.

Dr. Adams calls himself a zealot; and is willing to bestow the same compliment on Dr. Willan. I trust, however, that neither of those gentlemen will ever make good his title to that opprobrious epithet; nor on any occasion betray more zeal than discretion.

The world is much obliged to Dr. Adams for reminding us of Mr. Hunter's criterion of the small-pox; which, I trust, will in future supersede the practice of inoculating with matter suspected to be variolous; a practice to which some gentlemen are extremely fond of having recourse upon every occasion, regardless of the consequences it may produce. Thus the mischiefs arising from the failures, and even the supposed failures of vaccination, are multiplied a hundred fold.

In the last number of the Medical and Chirurgical Review is a contribution to vaccine literature, which, if not very learned or ingenious, is at least very long; it forms a part of a deep conspiracy against vaccination. The author tells us, that "speaking abstractedly," (he means *distractedly*), "it is of no moment in which way the question respecting the vaccine practice is ultimately determined." This is a most extraordinary assertion. The hopes of the public have been raised to the highest pitch; and after being taught to expect a mild substitute for the small-pox, and the extermination of that dreadful disease, we are told, it is of no consequence whether their hopes are realised or not.

We are also told, that the author of the article asked permission to consult the Minutes of the Vaccine-Pock Institution, in Broad Street, Golden Square; *who* had taken much pains to investigate the different cases. He obtained permission; and, after consulting the Minutes, appears to have made as free with them as if they were his own.

With respect to the Cases in Fullwood's Rents, we are informed the account received from Mrs. Hodges seems to prove, that the progress of vaccination was regular and satisfactory; and that the subsequent small-pox is not controverted. But in opposition to these, and other supposed failures, failures, we are afterwards told, that Dr. Pearson attributes them to the negligence and ignorance of inoculators; and endeavours to prove, by a comprehensive view of the subject, and a very copious induction of facts, *that notwithstanding the late failures, and many more that are to be expected, it is a law of the animal economy, to have the susceptibility of the small-pox totally destroyed by the cow-pock; and that there is no more reason to doubt the universality of this law in the inoculation of the cow-pock, than in the inoculation of the small-pox.*

This being admitted, it follows of course, that certain persons, either through ignorance, or self-interest, or malevolence, have spread false alarms; filling the minds of parents with groundless fears, and doing an injury to the rising generation, for which they can never make an atonement.

We next learn the result of inoculations instituted by Dr. Pearson, for the sake of ascertaining whether the cases in Fullwood's Rents were variolous. This, I trust, will be the last report of such experiments, the necessity of which can no longer be pleaded, nor the criminality of instituting them palliated, since the criterion pointed out by Dr. Adams is generally known.

In one case there was the regular small-pox; in another there were some eruptions resembling the tooth-rash, or such as occur frequently after the cow-pock. These, which on a former occasion I have termed *miliary eruptions,* are the effect of simple cuticular inflammation, produced by mere heat, or any other stimulus whatever; and are so common as not to excite much attention, unless they occur after putting a vaccine patient to the test of variolous inoculation. But in that case, mole-hills are turned into mountains, and miliary eruptions into the small-pox.

Several instances of this sort I have noticed on former occasions; and others are continually starting up like hobgoblins, spreading a panic among women and children, and a *pretended panic* among certain medical practitioners; some of whom cannot conceal their exultation on account of the recent failures, or their sanguine hope, that this branch of practice will in future be confined to professional men.

It is, however, a little surprising, that these gentlemen did not shew any signs of sorrow on this melancholy occasion. Had a series of similar occurrences realised their expectation, and confirmed their opinion, even in that case, the enthusiasm manifested by others in favour of vaccination vaccination would not have been an object of censure, but a fault—

Ignoscenda quidem, scirent si ignoscere manes.

I have, on former occasions, related instances of the small-pox occurring a second time. In the article under consideration we are told, that Dr. Woodville has never met with an instance of this kind; and Mr. Wachsel is doubtful whether he has or not. I shall, therefore, add others which have since come to my knowledge.

The first is that of Mrs. Smith, of Jennings's Row, Kensington, who was inoculated when she lived at Catfield, in Norfolk, by Mr. Downes, with recent matter taken from a child, who, together with her mother, laboured under the small-pox; of which the mother died.

Mrs. Smith was taken to the apartment where these variolous patients lay, and inoculated on the spot. Her arm rose, she sickened, and had about fifty pustules. She was then about nineteen years of age.

Above twenty years after, her daughter, who now lives with her, had the confluent small-pox in the natural way; in consequence of which she is much pitted. Mrs. Smith caught it of her, and had it so severely, that her husband counted about five hundred pustules on her back.

The second case is that of Mrs. Skittleworth, at No. 3, Horse Shoe Alley, York Street, Westminster; who was inoculated for the small-pox in Wales, when nine months old. The matter was taken from a child, who, together with its mother and three other children, was covered with the natural small-pox. The woman was so ill, as to be unable to suckle her child; it was, therefore, suckled by Mrs. Skittleworth's mother, together with her own. Mrs. S. has repeatedly been informed by her mother, that her arm rose, that she sickened, and had about twenty-five pocks. On coming to London, she caught the small-pox in the natural way, has had a very heavy burden, and is much marked by that cruel distemper.

Having asked this woman why she did not have her child inoculated, she answered, "Sir, I was inoculated myself in my infancy, when in Wales; and, on coming to London, caught the small-pox again, and had it as you see."

With respect to the cases in Fullwood's Rents, one of them was over before I heard of the affair; the other I acknowledged to be the small-pox at first sight. Whether either or both of them previously had the perfect and genuine genuine cow-pock, I am not quite so certain. The evidence in favour of that opinion is undoubtedly strong; and would be conclusive, had we not been taught by Dr. Jenner and other practitioners, that a spurious pustule, produced by vaccine virus taken at a late period, may so nearly resemble the genuine, as to be mistaken for it; and that such a spurious pustule is capable of perpetuating its like.

Since I first had reason to believe this hypothesis, I have cautiously abstained from taking matter at a late period; and am therefore not fully competent to decide the question; but if that hypothesis is well founded, it is not sufficient to assert, that the matter with which one of the children was inoculated, was taken on the eighth day, unless it can also be proved, that it had not been previously vitiated by a neglect of this rule. As to the other child, it is not known on what day the matter, with which she was inoculated, was taken.

I am, however, ready to admit, for the sake of argument, though by no means convinced of the fact, that both those children had the small-pox after the cow-pock. While failures in the inoculation of the small-pox are so numerous, it is not necessary to maintain the doctrine of those zealots in vaccination, who contend, that no failure can possibly occur in the inoculation of the cow-pock.

> Non tali auxilo, neque defensoribus istis,

> Tempus eget.

An objection has been urged against any testimonial, declaring that the cow-pock is a perfect security against the small-pox. The objection is indeed plausible, but not new. However, till better and more accurate testimonials are published, it may be sufficient to remark, that many who subscribe the present, only mean to lay down a general rule; which is not without exceptions. Nevertheless, it would be more correct, and more justifiable, to declare an opinion, that those who have had the cow-pock are as secure as those who have had the small-pox.

The Kensington case is again obtruded on the public, in the Medical and Chirurgical Review, and with no small degree of effrontery. A committee, consisting of Dr. Pearson, Mr. Payne, and Mr. Young, is said to have been appointed by the board of the Vaccine Pock Institution, to investigate this case; in which, it is justly observed, the question of the child's having had the cow-pock, is the only point in dispute.

Having

Having enquired into this case very minutely more than once as an individual, and afterwards as a member of a committee appointed by the Medical Council of the Royal Jennerian Society, I was so fortunate as to discover two very important circumstances, overlooked by those who had previously enquired into the case; some of whom were very anxious to prove it had been a case of cow-pock, in order to explode the practice; others, *because it occurred at a rival institution.*

The first of these circumstances was, *that the inoculator never saw the child after the fourth day*; the second, *that the pustules were both ruptured at an early period.* This latter circumstance, which rendered it more difficult to determine whether the disease had been genuine, and whether it went through its regular course, is carefully kept out of sight, in all the various and contradictory reports of the case, published in the Medical and Chirurgical Review.

Even in the present account, there is one blunder. The *mother* did not attend at the inoculator's house on the Monday following, but the *daughter.* This is not material to the point at issue; but it is material towards shewing the inaccuracy of the report.

As another proof of its inaccuracy, we are told the child's *arms deadened,* and became *an entire* scab. Mrs. Meredith had first declared the scab was *brown,* and afterwards that it was *black.* She now declared, that she could *neither say it was black or brown.*

The report concludes with observing, that Mr. and Mrs. M. appear to be much superior to others in the same rank of life, in consistency and accuracy. As a proof of this, we are told, he had kept minutes of the illness of the child in the small-pox, and regretted that he had not done the same in the cow-pock. Mr. M. is a shoemaker, and for his rank in life may be a sensible man, nay, he may be as good a scholar as the reporter of the case in the Review; it will, nevertheless, be adviseable for him to remember the old adage—Ne sutor ultra crepidam.

I cannot dismiss this subject without remarking, that had the father of lies been concerned in propagating a report concerning it, he could not more effectually have disguised the truth, than has in general been done on this occasion. It is evident that there is some individual, who is determined to prove the failure of vaccination in this instance; instance; and though repeatedly baffled in his attempt, returns again to the charge:

> Destroy the fib, and break the web,—and then
> The creature's at his dirty work again.

Such perseverance, in such a cause, carries with it rather an appearance of persecution; an appearance of envy, hatred, malice, and all uncharitableness, than of public spirit, and a love of truth:

> But what will not ambition and revenge
> Descend to?

Another case recorded in these reports, is one that occurred in my practice, in the year 1800. The subject of it was the daughter of Mr. Smith, at that time residing in Dorset Street, Salisbury Square; not in Wilderness Lane, her present residence, as stated in the report.

This error is of no consequence with regard to the grand point in question, which is the efficacy of the cow-pock; but it is of some consequence as far as it shews the careless and negligent manner in which this, as well as other cases in the report, is drawn up.

The pustules in this child were remarkably small, and bore no comparison to those of her sister, who was inoculated at the same time. The pustule, with the surrounding inflammation, did not exceed the size of a pea; and it dried and fell off so soon, that the arm was well in a fortnight after inoculation. In this respect also, the report in the Review is very incorrect; as any one may learn, who will take the trouble of calling on Mrs. Smith, at No. 34, Wilderness Lane.

No scar, resembling that of cow-pock, remains; and it is the opinion of every one who has seen the arms, that the attempt to vaccinate this child proved abortive. It will also appear, by inquiring of Mrs. Smith, that those persons who circulated a report of the child's having had the small-pox after the cow-pox, knew that they were circulating a falsehood.

The members of the Vaccine Pock Institution who communicated the case, were Dr. Pearson and Mr. Coxwell; who had visited the patient. The opinion of the officers of the Institution is, that she did not go regularly through the cow-pock. To this opinion I readily subscribe; and freely confess, that whatever blame is due, it ought to fall on the practitioner, and not on the practice.

I must, however, beg leave to state the following particulars, as the best apology for my error. At that time

I practised gratuitous inoculation of the cow-pock to a very considerable extent, both at home and abroad. Among others I inoculated the two children of Mr. Smith; first at his brother's house at Newington, and afterwards at my own house.

I then followed the rule laid down by certain inoculators, who pretended to know better than Dr. Jenner,—that of taking the vaccine fluid as long as it continued to be limpid; a rule which has proved fallacious in other instances, as well as in the present; but this is the only one, as far as I know, in which I did not detect its fallacy at the time.

The children of Mr. Smith were to have been brought to me again for inspection on a particular day; but when I called next at his brother's, I was informed it would not be convenient to their parents to bring them, on account of the distance. I therefore called once to see them; and should have called oftener, as, every one must know, ought to be done in all cases of this kind, when practicable; but other avocations prevented me. At the same time, I must own, that from the very imperfect knowledge of vaccination I then possessed, the cases appeared to me satisfactory.

I am content to bear the blame on this occasion; but am compelled, in my own defence, to mention the difficulties and disadvantages under which I then laboured. I was propagating the gratuitous inoculation of a disease scarcely known even by name, from house to house, through the large and populous district of Southwark, and the neighbouring villages; not only without the aid of medical practitioners, but in direct opposition to many of them, some of whom had recourse to the most illiberal and wicked arts, in order to check my progress. Having had such obstacles to encounter, it is to me a great consolation, that no more charges are yet brought forward against me in the same authentic way; and I shall be tolerably well satisfied, after passing the fiery ordeal now instituted, if twenty other similar cases should not rise up in judgment against me.

I am, &c.

*New Street, Hanover Square.* JOHN RING.

